# Self-Reported Anxiety in Spain: A Gendered Approach One Year After the Start of COVID-19 Pandemic

**DOI:** 10.3389/fpubh.2022.873891

**Published:** 2022-06-16

**Authors:** Constanza Jacques-Aviñó, Tomàs López-Jiménez, Matthew Bennett, Laura Medina-Perucha, Brenda Biaani León-Gómez, Anna Berenguera

**Affiliations:** ^1^Unitat Transversal de Recerca, Fundació Institut Universitari per a la recerca a l'Atenció Primària de Salut Jordi Gol i Gurina (IDIAPJGol), Barcelona, Spain; ^2^Universitat Autònoma de Barcelona, Barcelona, Spain; ^3^Independent Researcher, Barcelona, Spain; ^4^Departament d'Infermeria, Universitat de Girona, Girona, Spain

**Keywords:** anxiety, gender, COVID-19, health inequities, mental health, immigrants, sexual orientation, pandemic

## Abstract

The COVID-19 pandemic has an impact on mental health. However, there is little evidence on how different axes of social inequity influence mental health from a gender perspective and over time. Our aim is to analyze anxiety according to gender identity and other axes of social inequities (migration status, sexual orientation, age, and employment conditions) one year after the start of the COVID-19 pandemic in Spain. We conducted a cross-sectional study among adults living in Spain with an online survey from April 8 to May 28, 2021. The main variable was anxiety measured by Generalized Anxiety Disorder Scale (GAD-7). Sex-stratified multivariate logistic regression models were constructed to assess the association between axes of inequities and anxiety. Our findings (*N* = 2,053) suggest that women have greater anxiety risk than men (35.2 vs. 28.2%, respectively). We observe in both genders that there is a clear age gradient, with anxiety decreasing as age increases; and that there is an association between worsening employment status and anxiety risk, although there is a difference between women by education level. Additionally, not having Spanish nationality is also associated with greater anxiety risk in women. In men, identifying as non-heterosexual is associated with a higher risk of anxiety. The axes of inequities have different effects according to gender identity. These differences in anxiety risk by population subgroup must be taken into account in order to sensibly and equitably treat the surge in mental health disorders brought on by the COVID-19 pandemic.

## Introduction

Evidence shows that people of a low socioeconomic status have a disproportionately high risk of COVID-19 infection and that vulnerable communities have higher exposure to the rapid spread of the virus and a higher mortality (1–5). Moreover, the pandemic has worsened the financial situation of those with precarious employment conditions, whose financial margins were already at risk (6). Taking a gender perspective into account, women are more affected by this pandemic, as they are overrepresented in sectors that are most affected by the current crisis (e.g., retail, hospitality, care, and domestic work) (7). In addition, the burden of unpaid caregiving and work-life balance has fallen largely on women during periods of lockdown. Studies carried out in Europe show that women spent on average 62 h per week caring for children (compared to 36 h for men) and 23 h per week doing housework (15 h for men) (7). Findings suggest that these social inequities are related to worse mental health outcomes among women (8–10). Also, these gender-based inequities has been associated with increases in gender-based violence, impacts on the different phases of reproductive and maternal health, fear of infection and infecting others, as well as taking on caregiving tasks in complex contexts and being conscientious about the negative impact their own discomfort can have on their children (8, 11–13). However, there is a significant under-representation of women and gender diverse people in COVID-19 decision-making bodies, which may hinder the effective management of COVID-19 for these subgroups and act as a multiplier of pre-existing gender-based inequities (14, 15).

On the other hand, migration is a complex process which affects people differently. Migration often results in traumatic events, barriers and challenges which lead to increased prevalence of psycho-social and other health issues among migrant populations when compared to the local population (16). Studies that consider the mental health of migrant populations during the COVID-19 pandemic report that migrant populations faced an increased risk of COVID-19 infection due to crowded living conditions and employment type, both public-facing and informal (e.g., house cleaning). In fact, in our previous study conducted in Spain at the start of lockdown (between April and May 2020), the migrant population was more concerned (85.6%) about economic problems than people who were born in Spain (79.4%) (17). Furthermore, the suspension of public administration has led to delayed appointments for legal procedures, affecting numerous immigration and asylum procedures (18). In addition, migrant populations reported barriers to accessing COVID-19-related health services, such as language interpretation and fear of deportation due to legal status (19, 20). To this date, there are few studies in Spain that consider migration status in relation to gender and health during this period.

The COVID-19 pandemic has additionally impacted other social identities which have remained at the margins, as is the case for people who do not identify within the heteropatriarchal model (21–23). Previous evidence shows that adults and adolescents who identified as a sexual and/or gender minority reported elevated anxiety levels (24). Poorer mental health outcomes in these subgroups can be explained with a psychological framework, due to stigmatization and greater social and interpersonal problems compared to heterosexual individuals (25). Studies conducted during the pandemic show that adults identifying as sexual minorities are more likely to report living in households with food and economic insecurity than heterosexual respondents (26). In addition, over 52% non-heterosexual individuals experienced depression in the last year and a similar proportion had self-harmed or considered taking their own life (27). Thus, the COVID-19 pandemic could have mental health effects both in previously healthy people as well as in people with pre-existing mental health disorders (28).

The contributions of this study are as follows. Our interest is to study how the Spanish resident population is dealing with pandemic one year later, by the first quarter of 2021 taking into account a gender approach (29, 30). During this period, the country was still experiencing a series of mobility restrictions and shop closures. Using a gender perspective implies including different axes of inequality to highlight social disparities. According to previous studies, sexism, racism, homophobia, classism, and aging represent axes of oppression that manifest in different ways and worsen population health (31–33). According to the evidence these measures affect societies in three manners: (1) exposing existing vulnerabilities, (2) reinforcing current inequalities, and (3) amplifying social differences in the future because of scarring effects (34). Thus, the COVID-19 pandemic reveals disproportionate risk and impact based on structured inequality at intersections of ethnic minority status, gender and class, as well as occupation (ethnic minorities, women, and undocumented workers) (35).

As public health and primary care researchers, we strive to look at specific subgroups to identify mental health outcomes related to possible experiences of discrimination and inequality. We will focus on anxiety, given that it is a symptom that manifests itself in uncertain situations (36). Therefore, our research question is: *How are anxiety levels in Spanish residents one year after the first wave of COVID-19 pandemic? Which population subgroups are most affected psychologically, considering various axes of social inequality?* Our aim is to analyze anxiety levels according to gender identity and other axes of social inequality (migration status, sexual orientation, age, and employment conditions) one year after the start of the COVID-19 pandemic in Spain.

## Methods

The present study is part of a larger project in Spain, for which we conducted an online survey during the start of the COVID-19 pandemic and carried out other qualitative studies. We are also conducting this same study in several Latin American countries. This paper is a cross-sectional study among adults living in Spain using data collected from a second online survey. Data were obtained and maintained between April 8 and May 28, 2021 using REDCap (Research Electronic Data Capture), an electronic data capture tool hosted at Fundació Institut Universitari per a la recerca a l'Atenció Primària de Salut Jordi Gol i Gurina (IDIAPJGol). REDCap is a secure, web-based software platform designed to collect data for research studies, providing (1) an intuitive interface for validated data capture; (2) audit trails for tracking data manipulation and export procedures; (3) automated export procedures for seamless data downloads to common statistical packages, and (4) procedures for data integration and interoperability with external sources (37, 38).

Recruitment was done through online platforms, social media and contact with community-based organization around Spain using convenience sampling techniques. The survey was piloted with people with different sociodemographic characteristics. Both the research team and participants contributed to the recruitment and dissemination of the on-line survey, using snowball strategies. The average time to answer the survey was 10 min, which was specified on the front page of the survey. Data collection was finalized when the borders of the cities were opened and greater mobility for displacement was given within the autonomous communities in Spain. This study has been informed by gender perspective and the social determinants of health (39–41). All members of the research team are highly sensitive of the different axes of social inequities, and the multiple power structures that differentially impact the population unequally.

Our final study population was comprised only of people who identified with the gender binary, identifying either as a man or woman with regards to gender identity. We understand gender identity as a person's understanding and experience of their own gender (42). Participants were allowed to select one of the following options for gender identity: female, male, non-binary and other. People who selected the “non-binary” or “other” were excluded from the present analyzes, as they will be included in a future study that is exclusive to the LGBTQI+ community. The final study population included cisgender participants: people whose gender identity matched their sex at birth. All data were stratified by gender identity (women vs. men) for final analysis.

The main outcome was anxiety, which we measured using the Generalized Anxiety Disorder 7-item (GAD-7). The GAD-7 has several advantages: it is easy to use, it is a validated instrument, has clear psychometric properties, and consists of only seven items (43). Moreover, it is an instrument adapted to the Spanish population (44). Anxiety was defined as excessive worry and persistent restlessness related to different elements such as personal health, employment, social interactions and everyday life situations (36). The anxiety variable was categorized as normal, mild, moderate and severe. We considered an individual to have anxiety if they reported moderate or severe levels. Sociodemographic variables, including age, having Spanish nationality, education level, being an essential worker, employment and socioeconomic status, housing and living conditions, care work, concern for cohabitant, domestic violence, and perceived social support were considered as independent variables. We calculated absolute and relative frequencies of these sociodemographic variables, stratified by gender identity. Multivariate regression models were constructed to evaluate the association between anxiety variable and socio-demographics and social variables.

Differences between groups were assessed using the chi-square test, the Mann–Whitney *U*-test or the *t*-test. Box plots were used to chart the distribution of GAD-7 values in the different nationality, sexual orientation, age, study level and work condition groups, stratified by gender. Univariate and multivariate logistic regression models were constructed to evaluate the association between nationality, sexual orientation, age and employment conditions with anxiety (GAD-7). Crude and adjusted odds ratios (ORc & ORa) and 95% confidence intervals (95% CI) were calculated. Analyzes were stratified by gender identity. We have carried out four types of models for women and men. Model 0: crude models (one model for every variable); model 1: variables adjusted for nationality and sexual orientation; model 2: variables adjusted for nationality, sexual orientation and age; model 3: variables adjusted for nationality, sexual orientation, age, and employment status. The level of statistical significance was set at 0.05 and all tests were two-tailed. All analyzes were performed in Stata 17.0.

## Results

A total of 2,053 individuals were selected in the study, of which 73.4% identified as women, 26.6% as men. 5.8% of women and 6.8% of the men did not have Spanish nationality. 10.8% of women and 18.1% of men did not identify as heterosexual. The median age of women was 45 years (IQR: 35–54), vs. 48 years (IQR: 35–56) for men. 22.0% of women's employment conditions worsened since the start of the pandemic, compared to 27.6% of men. Detailed characteristics of the study population by gender identity are available in Table 1.

**Table 1:** Socio-demographic characteristics and social variables by gender identity (*n* = 2,053).

	**Women**	**Men**	**Total**	***P*-value^**a**^**
**GAD-7**				
Normal/Mild	976 (64.8%)	392 (71.8%)	1,368 (66.6%)	0.003
Mod/Severe	531 (35.2%)	154 (28.2%)	685 (33.4%)	
**Age**				
18–30 years	259 (17.2%)	100 (18.3%)	359 (17.5%)	<0.001
31–50 years	724 (48.0%)	208 (38.1%)	932 (45.4%)	
51–64 years	445 (29.5%)	183 (33.5%)	628 (30.6%)	
≥65 years	79 (5.2%)	55 (10.1%)	134 (6.5%)	
Median (p25–p75)	45 (35–54)	48 (35–56)	46 (35–55)	0.010^b^
Mean (SD)	44.8 (12.5)	46.5 (14.0)	45.2 (12.9)	0.007^c^
**Nationality**				
Spain	1,419 (94.2%)	509 (93.2%)	1,928 (93.9%)	0.433
Other countries	88 (5.8%)	37 (6.8%)	125 (6.1%)	
**Place of birth**				
Spain	1,339 (88.9%)	498 (91.2%)	1,837 (89.5%)	0.124
Other countries	168 (11.1%)	48 (8.8%)	216 (10.5%)	
**Sexual orientation**				
Heterosexual	1,344 (89.2%)	447 (81.9%)	1,791 (87.2%)	<0.001
Non-hetero	163 (10.8%)	99 (18.1%)	262 (12.8%)	
**Education level**				
University	1,245 (82.7%)	425 (78.0%)	1,670 (81.4%)	0.016
Non-university	261 (17.3%)	120 (22.0%)	381 (18.6%)	
**Work**				
Working/Retired	1,226 (81.4%)	427 (78.2%)	1,653 (80.5%)	0.112
Other	281 (18.6%)	119 (21.8%)	400 (19.5%)	
**Employment status**				
Not worsened	1,176 (78.0%)	396 (72.5%)	1,572 (76.6%)	0.009
Worsened	331 (22.0%)	150 (27.5%)	481 (23.4%)	
**Living with children**				
No	674 (53.2%)	293 (62.5%)	967 (55.7%)	<0.001
Yes	594 (46.8%)	176 (37.5%)	770 (44.3%)	
**Concerned school situation of minors**				
Little None	107 (20.7%)	44 (28.6%)	151 (22.5%)	0.040
Mod-quite-a lot	410 (79.3%)	110 (71.4%)	520 (77.5%)	
**Housework**				
Equitable	616 (50.7%)	315 (69.4%)	931 (55.7%)	<0.001
Mostly me	472 (38.8%)	44 (9.7%)	516 (30.9%)	
Another person	128 (10.5%)	95 (20.9%)	223 (13.4%)	
**Housing m** ^**2**^				
Less 50 m^2^	77 (5.1%)	30 (5.5%)	107 (5.2%)	0.726
50–100 m^2^	471 (31.3%)	161 (29.5%)	632 (30.8%)	
>100 m^2^	959 (63.6%)	355 (65.0%)	1,314 (64.0%)	
**Concerns co-existence**				
Little None	979 (77.2%)	358 (76.3%)	1,337 (77.0%)	0.700
Mod-quite-a lot	289 (22.8%)	111 (23.7%)	400 (23.0%)	
**Self-perceived health**				
Excellent/Good	1,224 (81.2%)	464 (85.0%)	1,688 (82.2%)	0.049
Regular/Bad	283 (18.8%)	82 (15.0%)	365 (17.8%)	
**Social support**				
None/Little	102 (6.8%)	38 (7.1%)	140 (6.9%)	0.847
Moderate/Much	1,397 (93.2%)	501 (92.9%)	1,898 (93.1%)	

*SD, Standard Deviation*.

a *Unless otherwise stated, p-value derived from Chi-Square test*.

b *Mann-Whitney U test*.

c *T-test*.

**Figure 1:**
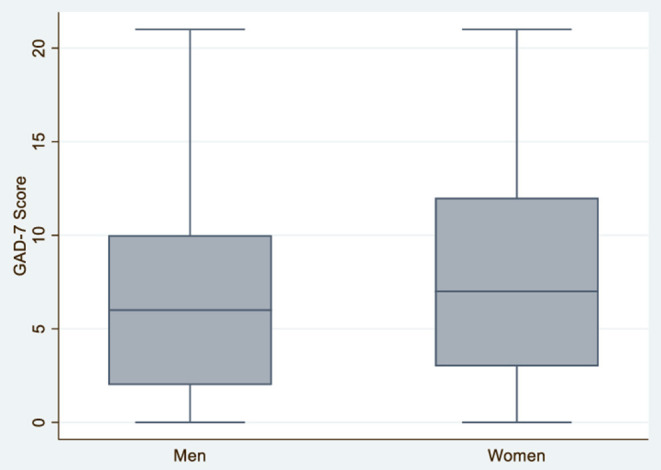
Anxiety according to gender identity in men and women *N* = 2,053.

The risk of anxiety was 35.2% in women and 28.2% in men (Table 1 and Figure 1). Women with a worse self-perceived health status reported higher levels of anxiety (Table 2). 61.5% of women who reported having poor self-perceived health had risk levels of anxiety, compared to 29.2% of women who reported having excellent health (*P* < 0.001). Women with high levels of social support reported lower levels of anxiety. For example, 33.7% of women with a high level of social support reported risk of anxiety, compared to 52.9% of women with no social support (*P* < 0.001) (Table 2). There was no statistically significant difference in anxiety levels among women who lived with minors, though women experienced higher anxiety levels associated with school performance. For instance, 40.7% of women who were very concerned about their child's performance at school reported moderate or severe anxiety levels, compared to 21.5% of women who expressed low concern (*P* < 0.001). Finally, there was no statistically significant difference in anxiety levels in women based on the amount of domestic work they undertook, 38.8% take care of household task alone and 50.7% share it.

**Table 2:** Association between sociodemographic characteristics, social variable, and anxiety (GAD-7)* among women and men in Spain (*n* = 2,053).

	**Women (*****N*** **=** **1,507)**			**Men (*****N*** **=** **546)**		
	**GAD-7**			**GAD-7**		
	**Normal/Mild**	**Mod/Severe**	***P*-value^**a**^**	**Normal/Mild**	**Mod/Severe**	***P*-value^**a**^**
**Age**						
18–30 years	155 (59.8%)	104 (40.2%)	<0.001	63 (63.0%)	37 (37.0%)	0.003
31–50 years	443 (61.2%)	281 (38.8%)		140 (67.3%)	68 (32.7%)	
51–64 years	313 (70.3%)	132 (29.7%)		142 (77.6%)	41 (22.4%)	
≥65 years	65 (82.3%)	14 (17.7%)		47 (85.5%)	8 (14.5%)	
Median (p25-p75)	46 (36–56)	43 (33–51)	<0.001	50 (38–58)	44 (31–52)	<0.001^b^
Mean (SD)	45.9 (12.8)	42.6 (11.7)	<0.001	47.9 (13.9)	42.9 (13.5)	<0.001^c^
**Nationality**						
Spain	929 (65.5%)	490 (34.5%)	0.022	367 (72.1%)	142 (27.9%)	0.554
Other countries	47 (53.4%)	41 (46.6%)		25 (67.6%)	12 (32.4%)	
**Place of birth**						
Spain	879 (65.6%)	460 (34.4%)	0.043	359 (72.1%)	139 (27.9%)	0.624
Other countries	97 (57.7%)	71 (42.3%)		33 (68.8%)	15 (31.3%)	
**Sexual orientation**						
Heterosexual	881 (65.6%)	463 (34.4%)	0.067	331 (74.0%)	116 (26.0%)	0.013
Non-hetero	95 (58.3%)	68 (41.7%)		61 (61.6%)	38 (38.4%)	
**Education level**						
University	826 (66.3%)	419 (33.7%)	0.004	307 (72.2%)	118 (27.8%)	0.631
Non-university	149 (57.1%)	112 (42.9%)		84 (70.0%)	36 (30.0%)	
**Work**						
Working/Retired	804 (65.6%)	422 (34.4%)	0.167	313 (73.3%)	114 (26.7%)	0.138
Other	172 (61.2%)	109 (38.8%)		79 (66.4%)	40 (33.6%)	
**Employment status**						
Not worsened	794 (67.5%)	382 (32.5%)	<0.001	306 (77.3%)	90 (22.7%)	<0.001
Worsened	182 (55.0%)	149 (45.0%)		86 (57.3%)	64 (42.7%)	
**Living with children**						
No	443 (65.7%)	231 (34.3%)	0.227	212 (72.4%)	81 (27.6%)	0.825
Yes	373 (62.8%)	221 (37.2%)		129 (73.3%)	47 (26.7%)	
**Concerned school situation of minors**						
Little-nothing	84 (78.5%)	23 (21.5%)	<0.001	36 (81.8%)	8 (18.2%)	0.163
Mod-quite-a lot	243 (59.3%)	167 (40.7%)		78 (70.9%)	32 (29.1%)	
**Housework**						
Equitable	407 (66.1%)	209 (33.9%)	0.560	242 (76.8%)	73 (23.2%)	0.018
Mostly me	297 (62.9%)	175 (37.1%)		28 (63.6%)	16 (36.4%)	
Another person	83 (64.8%)	45 (35.2%)		61 (64.2%)	34 (35.8%)	
**Housing m** ^**2**^						
Less 50 m^2^	42 (54.5%)	35 (45.5%)	0.002	13 (43.3%)	17 (56.7%)	0.001
50–100 m^2^	283 (60.1%)	188 (39.9%)		114 (70.8%)	47 (29.2%)	
>100 m^2^	651 (67.9%)	308 (32.1%)		265 (74.6%)	90 (25.4%)	
**Concerns co-existence**						
Little-nothing	682 (69.7%)	297 (30.3%)	<0.001	280 (78.2%)	78 (21.8%)	<0.001
Mod-quite-a lot	134 (46.4%)	155 (53.6%)		61 (55.0%)	50 (45.0%)	
**Self-perceived health**						
Excellent/Good	867 (70.8%)	357 (29.2%)	<0.001	354 (76.3%)	110 (23.7%)	<0.001
Regular/Bad	109 (38.5%)	174 (61.5%)		38 (46.3%)	44 (53.7%)	
**Social support**						
None/Little	48 (47.1%)	54 (52.9%)	<0.001	19 (50.0%)	19 (50.0%)	0.002
Moderate/Much	926 (66.3%)	471 (33.7%)		366 (73.1%)	135 (26.9%)	

*GAD-7 was categorized in Normal, Mild, Moderate and Severe.

*SD, Standard Deviation*.

a *Unless otherwise stated, p-value derived from Chi-Square test*.

b *Mann-Whitney U test*.

c *T-test*.

**Figure 2:**
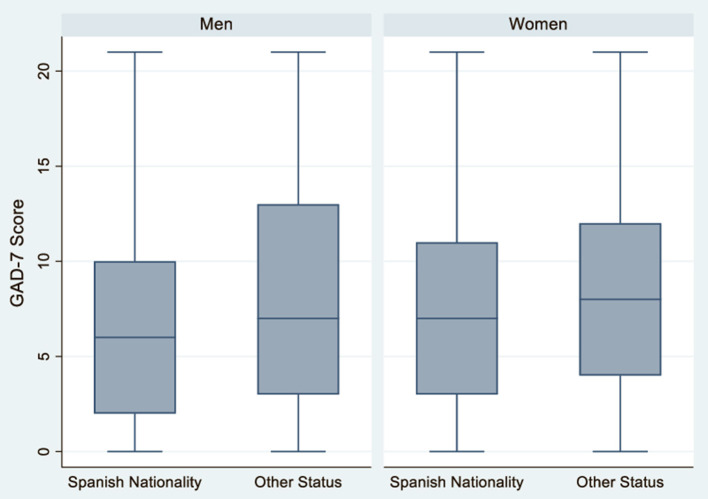
Anxiety according to nationality in men and women *N* = 2,053.

**Figure 3:**
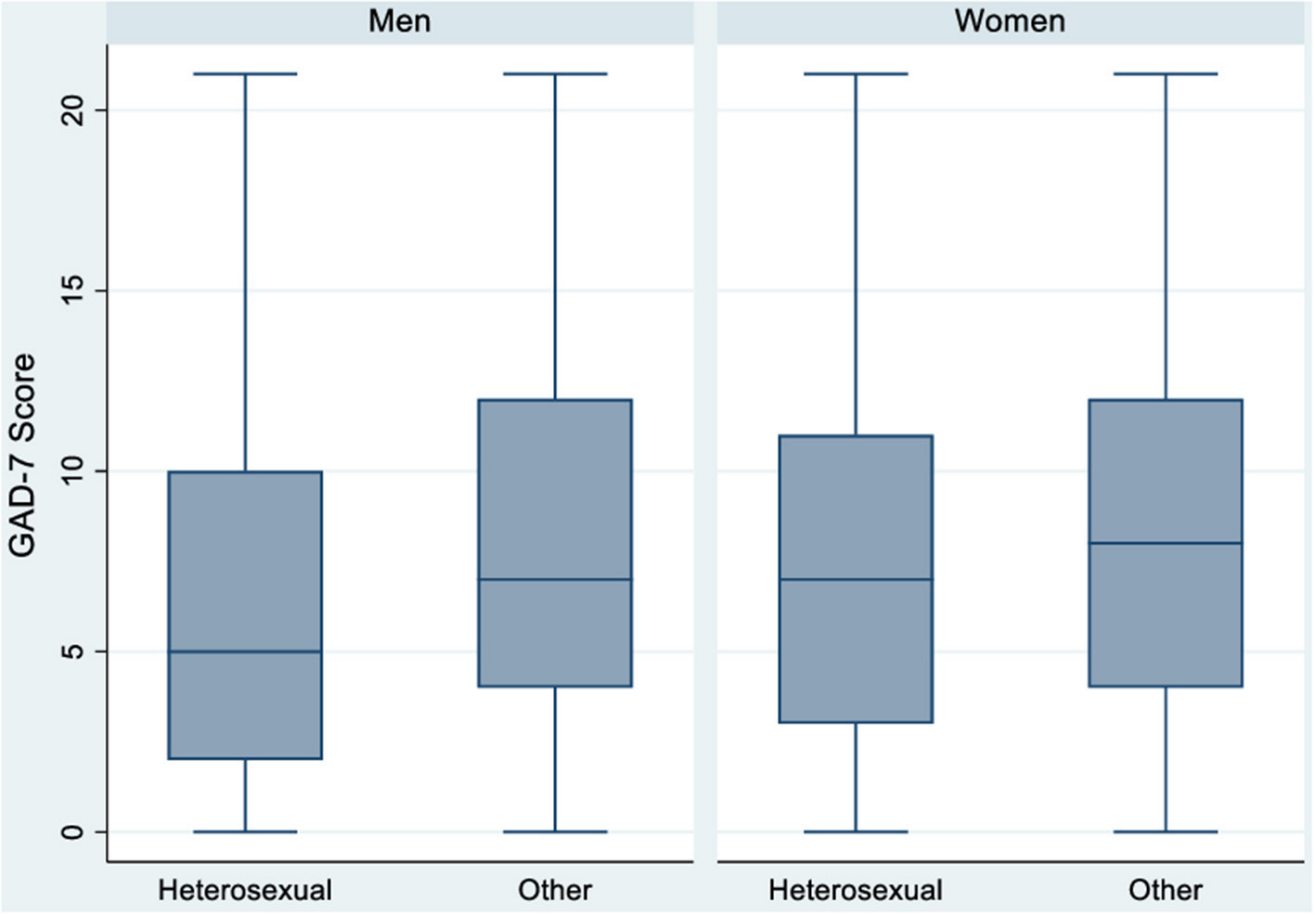
Anxiety according to sexual orientation in men and women *N* = 2,053.

**Figure 4:**
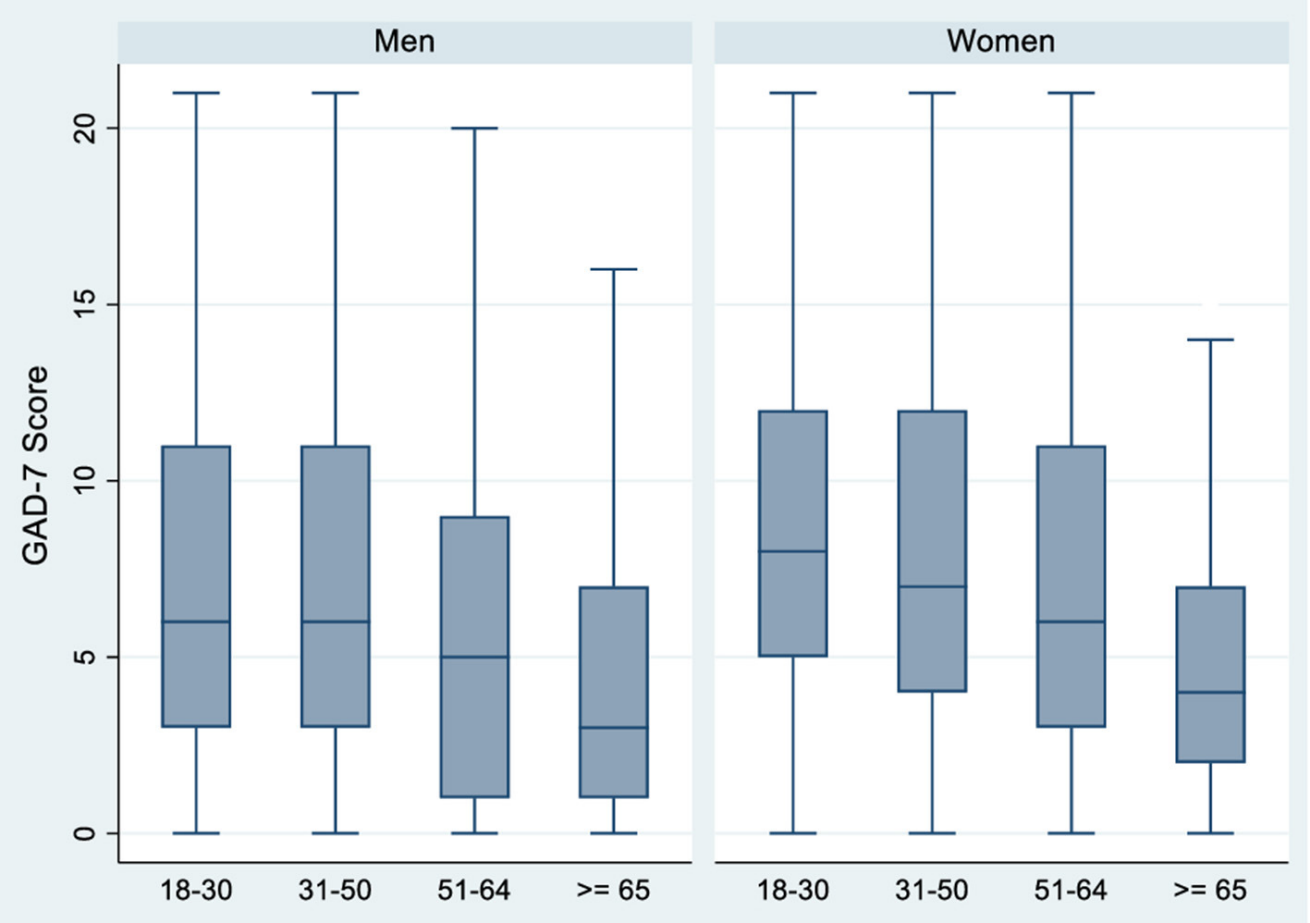
Anxiety according to age in men and women *N* = 2,053.

**Figure 5:**
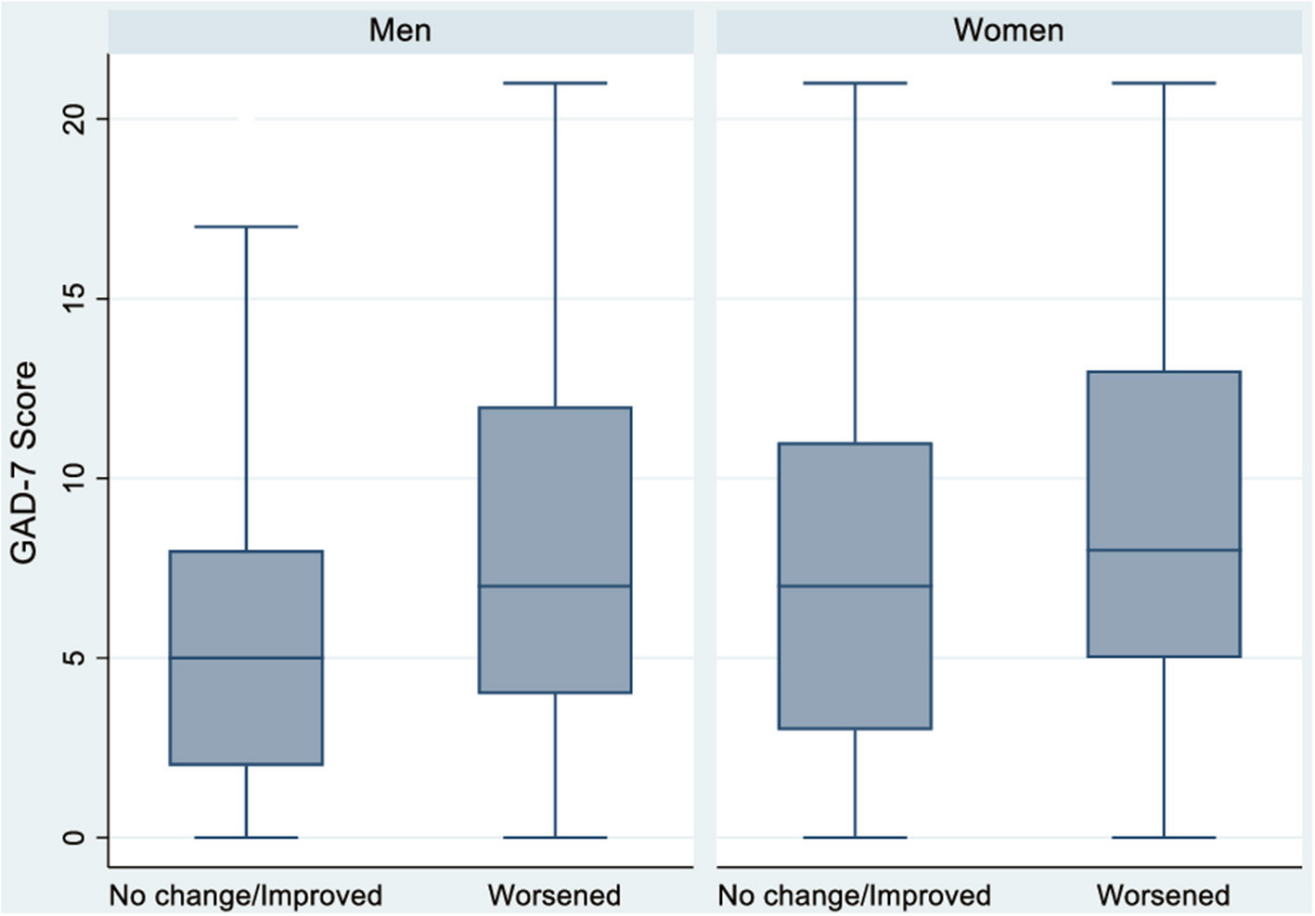
Anxiety according to work condition in men and women *N* = 2,053.

During the period of study, women without Spanish nationality experienced higher levels of anxiety than women with Spanish nationality (ORc = 1.65, CI 95%: 1.07–2.55) (Table 3A and Figure 2). Women without Spanish nationality continue to have a greater risk of anxiety when further adjusting the model for sexual orientation, age, and employment status, nationality, although the associations lose statistical significance (ORa = 1.36, 95% CI: 0.98–1.89). In women, there were no statistically significant differences in anxiety risk by sexual orientation in all models (Figure 3). However, anxiety risk decreased as age increased, with women aged 18–30 having the highest risk of anxiety (ORc = 3.12, 95% CI: 1.66–5.84; ORa = 3.01, 95% CI: 1.60–5.67) (Table 3A and Figure 4). With regards to employment status, women whose employment conditions worsened had a higher risk of anxiety across all models (Table 3A and Figure 5) than women whose employment status improved or remained the same (ORc = 1.70, 95% CI: 1.33–2.18).

**Table 3A:** Association between social axes of inequalities and anxiety (GAD-7) among women in Spain (*N* = 1,507).

	**Model 0**		**Model 1**		**Model 2**		**Model 3**	
	**ORc^**A**^ (95%CI)**	***P*-value**	**ORa^**A**^ (95%CI)**	***P*-value**	**ORa^**A**^ (95%CI)**	***P*-value**	**ORa^**A**^ (95%CI)**	***P*-value**
**Nationality**								
Spain	1.00		1.00		1.00		1.00	
Other countries	1.65 (1.07–2.55)	0.023	1.62 (1.05–2.50)	0.030	1.47 (0.95–2.29)	0.085	1.38 (0.89–2.16)	0.152
**Sexual orientation**								
Hetero	1.00		1.00		1.00		1.00	
Non-hetero	1.36 (0.98–1.89)	0.067	1.33 (0.96–1.86)	0.090	1.29 (0.92–1.81)	0.135	1.27 (0.91–1.78)	0.165
**Age**		<0.001^a^				<0.001^a^		<0.001^a^
65+	1.00	<0.001^b^			1.00	<0.001^b^	1.00	<0.001^b^
51–65	1.96 (1.06–3.61)	0.031			1.98 (1.07–3.65)	0.029	1.95 (1.05–3.60)	0.034
31–50 years	2.95 (1.62–5.35)	<0.001			2.88 (1.59–5.24)	0.001	2.78 (1.53–5.06)	0.001
18–30	3.12 (1.66–5.84)	<0.001			3.03 (1.62–5.70)	0.001	3.01 (1.60–5.67)	0.001
**Employment status**								
Not worsened	1.00						1.00	
Worsened	1.70 (1.33–2.18)	<0.001					1.63 (1.27–2.10)	<0.001

*ORc^A^, crude ordinal odd ratio*.

*ORa^A^, adjusted odds ratio*.

a *Wald test*.

b *Trend Test*.

Men with a worse self-perceived health reported higher levels of anxiety (Table 2). 53.7% of men with poor self-perceived health reported anxiety, vs. 23.7% of men with good or excellent self-perceived health (*P* < 0.001). Men with less social support experienced higher anxiety levels (53.4% of men with no social support vs. 23.7% of men with lots of social support reported risk of anxiety levels (*P* < 0.001). In contrast to women, there was no statistically significant difference in anxiety levels between men who lived with minors or men who were concerned about their child's academic performance. However, men who reported being primarily responsible for domestic work reported higher anxiety levels compared to men who either shared this work equally with another person or for whom another person took care of the domestic work. For instance, 36.4% of men who reported being primarily responsible for domestic care work reported anxiety, vs. 35.8% of men who did not perform any domestic work or 23.2% of men who shared this responsibility equally with another person (*P* = 0.018).

In men, there was no statistically significant difference in anxiety risk by nationality (Table 3B and Figure 2). However, non-heterosexual men had a higher risk of anxiety than heterosexual men (ORc = 1.78, 95% CI: 1.13–2.81). Non-heterosexual men have a greater risk of anxiety than heterosexual men across all models, though statistical significance is lost when further adjusting the model for sexual orientation, age, and employment status (ORa = 1.53, 95% CI: 0. 94–2.49) (Table 3B). As in women, anxiety risk decreased as age increased (Table 3B and Figure 4). Men aged 18–30 presented the highest risk of anxiety compared to the older age groups in all models (ORc = 3.45, 95% CI: 1.47–8.09; ORa = 2.34, 95% CI: 0.96–5.66). Finally, with regards to employment status, men whose employment worsened had a higher risk of anxiety compared to men whose conditions remained the same or improved, across all models (ORc = 2.53, 95% CI: 1.70–3.77; ORa = 2.44, 95% CI: 1.62–3.68) (Table 3B and Figure 5).

**Table 3B:** Association between social axes of inequalities and anxiety (GAD-7) among men in Spain (*N* = 546).

	**Model 0**		**Model 1**		**Model 2**		**Model 3**	
	**ORc^**A**^ (95%CI)**	***P*-value**	**ORa^**A**^ (95%CI)**	***P*-value**	**ORa^**A**^ (95%CI)**	***P*-value**	**ORa^**A**^ (95%CI)**	***P*-value**
**Nationality**								
Spain	1.000				1.00		1.00	
Other countries	1.24 (0.61–2.54)	0.555	1.10 (0.53–2.27)	0.804	1.07 (0.51–2.24)	0.852	1.01 (0.48–2.10)	0.988
**Sexual orientation**								
Hetero	1.00		1.00		1.00		1.00	
Non-hetero	1.78 (1.13–2.81)	0.014	1.76 (1.11–2.80)	0.016	1.48 (0.91–2.38)	0.111	1.53 (0.94–2.49)	0.088
**Age**		0.004^a^				0.014^a^		
65+	1.00	<0.001^b^			1.00	0.002^b^	1.00	0.005^b^
51–65	1.70 (0.76–3.96)	0.210			1.62 (0.71–3.71)	0.255	1.29 (0.55–2.99)	0.559
31–50 years	2.85 (1.28-−6.37)	0.011			2.64 (1.18–5.94)	0.019	2.20 (0.97–5.00)	0.059
18–30	3.45 (1.47–8.09)	0.004			3.01 (1.26–7.18)	0.013	2.34 (0.96–5.66)	0.060
**Employment status**								
Not worsened	1.00						1.00	
Worsened	2.53 (1.70–3.77)	<0.001					2.44 (1.62–3.68)	<0.001

*ORc^A^, crude ordinal odd ratio*.

*ORa^A^, adjusted odds ratio*.

a *Wald test*.

b *Trend Test*.

## Discussion

Our study has described the relationship between anxiety and gender one year after the start of the COVID-19 pandemic in Spain, while also taking into account other axes of social inequity. According to our results, women had a higher risk of anxiety than men during this period (35.2 vs. 28.2%, respectively). These proportions are higher compared to the same study conducted a year earlier in which 31.2% of women and 17.7% of men reported having anxiety (8). We observed four main findings: (1) in both genders there is a clear age gradient, with anxiety decreasing as age increases; (2) an association between worsening financial situation and anxiety, although there is a difference between women by education level; (3) in women, anxiety is also associated with not having Spanish nationality; and (4) in men, not identifying as heterosexual is associated with a higher risk of suffering from anxiety. We can see that the axes of oppression differ by gender identity.

In addition, anxiety risk is greater in women related to living in smaller spaces, concerns about coexistence with household members, poorer self-perceived health and the perception of not having adequate social support in case of need. Thus, access to safe and stable housing during lockdown is important, as it is a mechanism to ensure security and thermal comfort (45, 46). Our findings indicate that physical space and cohabitation have been especially relevant during this period, especially for women, due to the obligation to live together and the possible problems that could emerged in an uncertain social context. In previous studies, it has been found that women experience greater feelings of stress, dissatisfaction and lack of autonomy as they try to reconcile their self-identity with their imposed roles during the initial outbreak period (47). With regard to self-perceived health, previous studies have found an association between social class, psychosocial and physical working conditions and job insecurity and self-perceived health in men (48). Among women, there is an association between self-perceived health and low socioeconomic status, working conditions, material wellbeing at home and having little or no help with domestic work (48, 49). Likewise, in our study, the perception of having social support (i.e., feeling that friends, family or neighbors care about you) is a protective factor against anxiety. Several studies have shown the importance of the environment in the case of needing help, especially when professionals are not available (50–52).

We observed that the younger population (18–30 years) had higher anxiety levels compared to the older population (65 years or more). These results are consistent with the study we carried out during the lockdown of the first year of the pandemic (8). Although there are few studies on young people and social isolation, it is known that deprivation of social needs in this age group can have lasting effects on social interaction (53). On the other hand, it has been shown that studying online, experiencing tensions with family and increased exposure to social media can contribute to worsened mental health (54, 55). These emotional problems are exacerbated in families of a low socioeconomic status and could have long-lasting effects (56). This suggests that the COVID-19 pandemic should take into account age-specific needs, with a particular focus on the consequences of emotional problems during childhood and adolescence and their future health outcomes. Therefore, intersectoral action between families, primary care and the education system (among those who are in school) must be taken to address this (56).

Another finding is the association between higher levels of anxiety and worsening financial status since the onset of the pandemic, both in men and women. However, our study observed that not having university studies was a risk factor only in women. These results are consistent with studies conducted in Spain prior to the pandemic (57). Despite increased female participation in the workforce, women and men continue to be concentrated in economic activities deemed appropriate for their gender identity, bodies and social roles (31). Because women tend to have less education, this increases their vulnerability and risk of having worsened mental health outcomes as a result of unequal job opportunities (47). On the other hand, we did not observe any difference between being employed with a contract or having a pension and being unemployed or studying in our study population. These results coincide with studies at European level which have found that having a job in and of itself is not a protective factor, but rather depends on the quality and conditions of the job. In other words, having precarious work conditions negatively impacts mental health (58). Therefore, this leads us to conclude that having a job is not a protective factor for mental health, but depends primarily on the quality of working conditions.

In addition, we observed the association between migration status and anxiety. In our study, being in a permanent legal situation (e.g., having Spanish nationality) where one is not actively dealing with immigration paperwork or having to depend on immigration offices or job offers to renew residency permits is a protective factor. On the contrary, women who did not have Spanish nationality, despite being mostly in a legalized administrative situation, were more likely to have symptoms of anxiety. This association was not observed in men. In fact, migrant women, especially young women, have the highest unemployment rate in Spain (18). Consistent with other studies, poor living conditions due to low-income levels may have made it harder to respect curfews or work from home (59). Hegemonic structure such as immigration law affects people, especially in employment systems, as well as through government policies and actions (5, 60). Thus, limited access to the job market, immigration offices and social resources during the pandemic may explain these results in immigrant women.

Another key finding is the association between higher anxiety levels and identifying as non-heterosexual, observed only in men. This can be explained by the fact that in a heteropatriarchal society that assumes and imposes heterosexuality, non-dominant identities experience greater oppression (61). In a misogynistic culture, male homosexuality receives more social punishment, as it is associated with feminine behavior (62). Men are socially expected to subscribe to a model of male hegemony which includes certain behaviors that are not judged in the same way as women, such as being strong, taking risks and having many sexual partners (61, 63–66). From an anthropological approach, homosexuality is considered taboo for men, while being a “whore” is taboo for women, evidenced by the fact that using these attributes as an insult is a mechanism of social control (67). In fact, in a health survey conducted in Barcelona found that gay and bisexual men had a 185% higher likelihood of having anxiety/depression, while no difference was found in women with same-sex attraction (68).

## Limitation

In our case, the major limitation is the type of sample; we have primarily accessed one population (those who were able to respond to the survey, who we know are not the most vulnerable) to the detriment of other subgroups (69). As a result, our study is not entirely representative of the general population. Furthermore, we recognize our limitation of treating populations without delving into the heterogeneous identities of each of the groups that could explain different axes of oppression. Finally, we believe in the importance of directly involving people who are suffering from a health problem in the research processes and creation of health interventions that directly affect them (70, 71). Lack of comprehensive representation is one symptom of a broken system in which governance is not inclusive of gender identity, geography, sexual orientation, migration status, socioeconomic status or disciplines within and beyond health—ultimately excluding those who offer unique perspectives and expertise (15). Therefore, it is important to understand epidemiology, social science, biomedicine and citizen science as inherently political and as tools that explicitly works toward advancing social justice (50, 71, 72). It is thus important to reflect on the impacts of COVID-19 from a syndemic perspective, meaning that future studies must explore the different effects that the pandemic has on society while considering the intersection with other diseases and with the social conditions and axes of inequities that people experience (73). Intersectionality, a critical theoretical framework, provides a prism through which to examine the differential effects of COVID-19 across axes of social inequity. Intersectionality highlights how power and inequality are structured differently for groups, particularly historically oppressed groups, based on their varied interlocking demographics. Public health officials have told us that “we all” must be socially distant in order to flatten the curve, which has exacerbated social isolation, pain, and suffering. However, already marginalized populations have been forced to largely bear the burden of this suffering (35).

## Conclusion

After a year of the social and health crisis caused by COVID-19 in Spain, anxiety levels tend to increase in women and men, but with more severe outcomes in women. We believe that these effects on population mental health could last for a long time. Even if incidence and mortality rates were controlled, the experiences that produce suffering at particular points in time are carried throughout the life course. Factors such as age, gender, migration status and sexual orientation have a differential impact on anxiety in the population. Therefore, an interdisciplinary approach must be taken to combat the effects of the COVID-19 pandemic. The inequalities that existed before the appearance of COVID-19 have worsened in Spain, but differentially so according to social identity in women and men. Results that allow the idea of syndemia to be used to study and make visible different health outcomes other than COVID-19 alone. Policy makers at different levels of public administration should open a space for dialogue with citizens and the different sciences to assess which structural and social determinants work as a risk or protective mechanism for mental health.

## Data Availability Statement

Data cannot be shared publicly because of ethical restrictions. The Ethics Committee does not allow us to share the data publicly as our data contain sensitive personal information and cannot be fully anonymized. Data are available from the Research Ethics Committee of the Institut de Recerca en Atenció Primària Jordi Gol i Gurina (IDIAPJGol) (contact *via* cei@idiapjgol.info) for researchers who meet the criteria for access to confidential data.

## Ethics Statement

The studies involving human participants were reviewed and approved by 20/063-PCV. The submission of the answered questionnaire was considered to be their consent to participate in the study.

## Author Contributions

CJ-A: conceptualization, methodology, visualization, and writing—original draft. TL-J: conceptualization, methodology, data curation, formal analysis, visualization, and writing—review and editing. MB, LM-P, and BL-G: conceptualization, methodology, visualization, and writing—review and editing. AB: conceptualization, methodology, writing—review and editing, and funding acquisition. All authors contributed to the article and approved the submitted version.

## Funding

This work was supported by Spain's Ministry of Science and Innovation through the Carlos III Health Institute and European Union ERDF funds (European Regional Development Fund) through the Research Network in Preventive Activities and Health Promotion in Primary Care (redIAPP, RD16/0007/0001).

## Conflict of Interest

The authors declare that the research was conducted in the absence of any commercial or financial relationships that could be construed as a potential conflict of interest.

## Publisher's Note

All claims expressed in this article are solely those of the authors and do not necessarily represent those of their affiliated organizations, or those of the publisher, the editors and the reviewers. Any product that may be evaluated in this article, or claim that may be made by its manufacturer, is not guaranteed or endorsed by the publisher.
